# Therapeutic Potential of *Cynara scolymus* Extract and *Bifidobacterium longum* in Alleviating Diabetes-Induced Male Reproductive Dysfunction

**DOI:** 10.1155/ije/5884930

**Published:** 2025-10-19

**Authors:** Hadis Bozorgpoursavadjani, Haniyeh Keyghobadi, Delaram Moghadam, Reza Zare, Farshad Dehghani, Iman Jamhiri, Farhad Koohpeyma, Sanaz Dastghaib

**Affiliations:** ^1^Department of Biology, Zarghan Branch, Islamic Azad University, Zarghan, Iran; ^2^Autophagy Research Center, Department of Biochemistry, School of Medicine, Shiraz University of Medical Sciences, Shiraz, Iran; ^3^Student Research Committee, Shiraz University of Medical Sciences, Shiraz, Iran; ^4^Molecular Dermatology Research Center, Shiraz University of Medical Sciences, Shiraz, Iran; ^5^Endocrinology and Metabolism Research Center, Shiraz University of Medical Sciences, Shiraz, Iran; ^6^Autophagy Research Center, Shiraz University of Medical Sciences, Shiraz, Iran

**Keywords:** diabetes, *Hsd17b3*, spermatogenesis, *Star*, *Cyp11a1*

## Abstract

**Background:**

Diabetes mellitus (DM) is associated with a number of adverse effects on male reproductive health, including oxidative stress, testosterone deficiency, and spermatogenesis defects. Natural and probiotic-based therapies have gained popularity as a means of alleviating DM-related complications.

**Methods:**

Streptozotocin (60 mg/kg) was employed to induce diabetes in male Sprague-Dawley rats, which were then divided into eight groups, each receiving hydroalcoholic *Cynara scolymus* (Cynara) extract, *Bifidobacterium longum* (BBL) probiotic or a combination of both. The following parameters were measured: Fasting blood sugar (FBS), serum malondialdehyde (MDA), serum sexual hormones, and testicular MDA and total antioxidant capacity (TAC), as well as mRNA expressions of steroidogenesis enzymes *Star*, *Cyp11a1*, and *Hsd17b3*. Histopathological study and modified Johnson scoring system were performed.

**Results:**

Rats with diabetes treated with *Cynara*, *BBL*, and their combination showed a substantial reduction in FBS and MDA levels as well as an increase in TAC when compared to the diabetic group. The combined treatment demonstrated a greater elevation in serum luteinizing hormone (LH), follicle-stimulating hormone (FSH), and testosterone levels in comparison to individual treatments. The mRNA expressions of *Star*, *Cyp11a1*, and *Hsd17b3* increased significantly in the combined treatment group. Histological assessments revealed improved testicular architecture and germ cell populations in the combination therapy group.

**Conclusion:**

*Cynara* and *BBL* treatments in diabetic rats in combination produced a powerful protective effect on male reproductive function. The results showed that *Cynara* and *BBL* could be considered as promising therapeutic agents able to reduce the detrimental effects of diabetes on male reproductive health. Further research is needed to define their precise mechanisms and potential clinical uses.

## 1. Introduction

Diabetes mellitus (DM) is thought to be one of the primary risk factors for testosterone deficiency in men worldwide as a rapidly increasing chronic disease [1]. According to a WHO report in 2014, it is estimated more than 422 million adults worldwide suffer from DM [2]. The level of testosterone in men with DM is lowered by 25%–50%. It is also associated with increased mortality as well as insulin resistance [3]. Furthermore, poor sperm quality and infertility in diabetic patients are regarded as distressing [4].

Due to oxidative stress brought on by hyperglycemia, spermatogenesis, Leydig cell function, and reproductive cells are affected. Decreased libido and erectile dysfunction are associated with hormonal abnormalities, including decreased testosterone levels and altered gonadotropin production. Chronic hyperglycemia causes vascular disease that reduces blood supply to the reproductive organs, leading to food deprivation and hypoxia. Furthermore, testicular function is disrupted by diabetes-induced inflammation, which is characterized by increased proinflammatory cytokines. Diabetes-related neuropathy also impairs neuronal control that is essential for healthy reproduction, which can result in problems with ejaculation and erection. Finally, lower testosterone levels and sperm count are caused by increased apoptosis of Leydig cells and germ cells, which negatively affects diabetic patients' reproductive health [5–7].

Patients with DM develop high blood sugar levels, which can cause overproduction of reactive oxygen species (ROS). The influence created by ROS in patients with DM affects several signaling pathways and gene expression patterns [8]. If ROS levels are too high, or if oxidative stress conditions persist for a prolonged period of time, steroidogenic enzymes activity is reduced, which subsequently leads to a reduction in levels of testosterone [9]. Luteinizing hormone (LH) and follicle-stimulating hormone (FSH) are pituitary hormones with important roles in spermatogenesis. Antioxidant/oxidant imbalance ruins the hypothalamic-pituitary gonadal hormonal cross-talk, thereby ultimately resulting in a testosterone production decrease. Moreover, poor insulin production in diabetic rats modulates spermatogenesis, as it controls LH and FSH levels. LH triggers spermatogenesis by stimulating the expression of steroidogenic-related enzymes [10]. Steroidogenic acute regulatory protein (*Star*), 17-beta hydroxysteroid dehydrogenase 3 (*Hsd17b3*), and cytochrome P450 side chain cleavage enzyme (*Cyp11a1*) are involved in testosterone production. *Cyp11a1* and *Star* gene expressions induce the initiation step of steroidogenesis, whereas the *Hsd17b3*-derived enzyme directly produces testosterone from androstenedione [11]. Moreover, studies have indicated an inverse association between blood FSH levels and the risk of diabetes [12].

Currently, the most accepted treatments include insulin therapy and hypoglycemic medicine, though they have many unwanted side effects [13, 14]. Animal studies have shown that the administration of exogenous insulin leads to a decrease in weight of testicles and impairment of spermatogenesis [15]. Additionally, obese men with insulin resistance have lower levels of gonadotropins and testosterone, altered ratios of androgens to estrogens, abnormal semen parameters, and erectile function problems [16]. Meanwhile, there has been an increased interest in natural and probiotic treatments that may help counteract adverse impacts of DM [17–19]. Probiotics refer to live microorganisms orally administered, which enhance the intestinal health by altering the integrity of the intestinal microbiota [20]. A systematic review with a meta-analysis of relevant studies on the role of probiotics in DM parameters revealed that they can reduce HbA1c, fasting blood sugar (FBS), and also insulin resistance levels in Type 2 DM patients [17]. Probiotics, if consumed in an adequate quantity, can function to modulate the human gut microbiome, thereby directly influencing overall health through a largely unclear mechanism [21]. *Bifidobacterium longum* (BBL), a well-established probiotic strain, has exhibited promising hypoglycemic effects in Type 2 DM [22].

*Cynara scolymus L* (*Cynara*), commonly known as artichoke, is a perennial plant revered for its medicinal properties in the Mediterranean and Central Asia. Previous research has demonstrated the beneficial effects of *Cynara scolymus L* extract in ameliorating conditions related to metabolic syndrome, DM, and oxidative stress damages by improving the gut microbiome [23]. *Cynara* extract contains nondigestible oligosaccharides named inulin, which is known as a potential prebiotic owing to its high degree of polymerization [24]. Previous studies have highlighted BBL strains' capability to degrade the long-chain inulin found in *Cynara* [25]. A recent study has confirmed the potential antioxidant activity of symbiotic treatments in diabetic rats, as evidenced by the reduction in serum malondialdehyde (MDA) levels [26]. It has also been reported that the ingestion of two probiotics, BBL and *Lactobacillus rhamnosus* for 6 weeks in men with asthenozoospermic condition, elevated sperm motility, reduced DNA fragmentation, and lowered the H_2_O_2_ level as a result [27].

In the current study, we investigated the effects of a hydroalcoholic extract of *Cynara scolymus* L and BBL probiotics, both separately and in combination, on the expression of testicular steroidogenesis enzymes, testicular stereological characteristics, as well as parameters associated with obesity and diabetes in diabetes-induced Sprague-Dawley male rats.

## 2. Materials and Methods

### 2.1. Chemicals and Equipment

Streptozotocin was procured from Sigma (USA) (Catalog Number: S0130). The enzyme-linked immunosorbent assay (ELISA) kits, utilized for the assessment of rat LH, FSH, and testosterone, were sourced from the Bioassay Technology Laboratory (China) (Catalog Numbers: LH: E0179Ra, FSH: E0182Ra, Testosterone: E0132Ra). Kiyanzol isolation reagent, essential for RNA extraction, was obtained from KiyanDanesh (Shiraz, Iran) (Catalog Number: KDN2010). The cDNA synthesis kit (K1622) used in this study was acquired from Fermentas (Waltham, Massachusetts, USA). The SYBR Green PCR Master Mix Reagent Kit necessary for the polymerase chain reaction (PCR) analysis was provided by the ABI Company (Foster City, California, USA) (Catalog Number: 4309155), while the PCR Master Mix used was supplied by Amplicon (California, USA) (Catalog Number: A140303).

### 2.2. Animals

Ninety-six male Sprague-Dawley rats, aged 7–8 weeks, randomly allocated into eight groups, each consisting of 12 rats, were acquired from the animal laboratory of Shiraz University of Medical Sciences. Throughout the study, all the rats received a standard rodent diet and unlimited access to water. For one week preceding the commencement of the study, the rats were maintained under a 12-h light/dark cycle in a relative humidity level of 40%–60%, in accordance with standard laboratory protocols. The study received approval from the Institutional Animal Ethics Committee. The Research Ethics Committee of the Islamic Azad University–Arsanjan Branch has ethically confirmed the study (Ethics Code: IR.IAU.A.REC.1402.060).

### 2.3. Hydroalcoholic *Cynara* Extraction

*Cynara scolymus L* (Herbarium number 5167), which was initially identified as a biosystemic herb, was extracted in Natural Resources Department of the Hamedan Province Agricultural and Natural Resources Research Center. The hydroalcoholic extract was prepared by first weighing 100 g of dry powdered *Cynara*, followed by the addition of 500 mL of ethanol (70%). This mixture was soaked in a percolator for 72 h. Then, the obtained extract was concentrated using a rotary evaporator. Subsequently, the concentrated extract was subjected to drying in a desiccator connected to a vacuum pump, resulting in the collection of a dried brown powder extract. The extraction efficiency was 18.28/100 g. The solution obtained from the previous step was poured into a Petri dish and autoclaved. It was then maintained at a temperature below 50°C in dry, sterile conditions. Before beginning the treatment, 4 g of the dry extract was dissolved in 50 cc of physiological serum, creating a solution with a concentration of 80 mg/mL. The animals were administered this solution via gavage at a dosage of 400 mg/kg body weight. For example, in 200 g rats, 1 cc of solution was administered [28, 29].

### 2.4. Preparation of BBL Probiotic

Freeze-dried cultures of BBL (B. Christian Hansen (Denmark) supplied the strain longum (UABI-14). The lyophilized powder was inoculated to reach densities of 2–4 × 10^9^ CFU/mL, and it was subjected to centrifugation for cell manufacture of the BBL probiotic solution. Saline was used to suspend the isolated cells at a concentration of 1 × 10^9^ CFU/mL [29, 30].

The strains were inoculated into MRS broth and cultured in a 37°C incubator for 48 h. Then, 2% (v/v) was inoculated into the MRS (De Man–Rogosa–Sharpe) broth medium and cultivated for 24 h as an activated strain for use in the experiments until its light absorption at 600 nm reached 0.7 to 1. The samples were then centrifuged at 2500 rpm for 10 min, washed three times, and suspended at the proper cell concentration. Normal saline was used to suspend the isolated cells at a concentration of 1 × 10^9^ CFU/mL. The bacterial solution was adjusted daily in 10-mL vials for oral administration, ensuring that the rats received 10^9^ CFU/mL probiotics each time.

### 2.5. Experimental Design

To induce diabetes in 4 diabetic groups, a single intraperitoneal injection of freshly prepared STZ solution in 0.1 M citrate buffer (60 mg/kg, pH 4.5) was administered to the rats [14, 31]. Seven days after STZ injection, blood samples were collected from the tail veins of the rats to measure FBS levels. Rats with FBS levels exceeding 300 mg/dL were categorized into the diabetic group. The control group received no treatment.

1. The sham group received the equivalent volume of the NaCl 0.9% solution but did not receive any treatment.
2. The diabetic control group included diabetic rats that received the same volume of the solvent but did not undergo any treatment.
3. Experimental group 1 (*Cynara*) consisted of healthy rats treated with a hydroalcoholic extract of *Cynara* (400 mg/kg) [28].
4. Experimental group 2 (BBL) included healthy rats that were administered BBL treatment (1 mL^−1^ × 10^9^ CFU) [30].
5. Experimental group 3 (DM + *Cynara*) encompassed diabetic rats treated with *Cynara* hydroalcoholic extract (400 mg/kg).
6. Experimental group 4 (DM + BBL) involved diabetic rats that received BBL treatment (1 mL^−1^ × 10^9^ CFU).
7. Experimental group 5 (DM + BBL + *Cynara*) was composed of diabetic rats that received treatment with both the hydroalcoholic extract of *Cynara* (400 mg/kg) and BBL (1 mL^–1^ × 10^9^ CFU).

All the treatments were administered orally by gavage every morning for 48 days [32, 33]. At the beginning of the experiment, 12 animals were selected for each group. However, due to the long treatment period and mortality, 6 rats were ultimately selected and evaluated in each group. Upon the completion of the 48-day treatment period, the rats were anesthetized using a ketamine (10%) and xylazine (2%) mixture at a dose of 80/5 mg/kg (Alfasan, the Netherlands). Subsequently, 5 mL of blood samples was taken from the heart of rats as well and centrifuged for 10 min at 3500 rpm to isolate the serum, which was then stored at −70°C for further assessment of biochemical parameters. In this study, rats were fasted for 6–8 h before blood samples were obtained to measure FBS and other biochemical parameters.

After slaughtering the rats, the testes were isolated and weighed. The gonadosomatic index (ơ) was calculated by using formula that determined by testis weight divided by body weight × 100% [34]. Then, one of the testis tissues from each rat was preserved in a liquid nitrogen (N_2_) tank to maintain the RNA content. Another testicle was collected and fixed by a 10% formaldehyde solution for subsequent analysis.

### 2.6. Assessment of Serum Biochemical Parameters

Serum FBS is measured using biochemistry auto analyzer (Hitachi Japan) kits from the Bio-System Assay Company (Spain). The analysis of LH, FSH, and testosterone levels in the serum samples was performed using rat-specific ELISA kits (Bioassay Technology Laboratory, Shanghai, China). The tests were performed according to the manufacturer's specifications. Samples and standards were incubated with anti-LH, anti-FSH, and anti-testosterone in a 96-well plate with the antibodies coating. In the last step, the absorbance of the content of the wells was measured with an ELISA reader at certain wavelengths. LH: 0.5–100 mIU/mL detection range, 0.31 mIU/mL sensitivity, CV < 10% for intra-assay and CV < 12% for interassay. FSH; detection range: 0.2–60 mIU/mL; sensitivity: 0.022 mIU/mL CV < 10% for intra-assay and CV < 12% for interassay testosterone: sensitivity: 0.043 ng/mL; standard curve range: 0.1–40 ng/mL CV < 10% for intra-assay and CV < 12% for interassay.

Furthermore, the serum samples were also examined for MDA levels. MDA serves as an indicator of lipid peroxidation and was measured using a thiobarbiturate (TBA) reactive colorimetric method. To perform the experiment, 200 μL of collected serum was added to 800 μL of cooled 1.15% KCl. Then, 500 μL of this mixture was added to 3 mL of 1% phosphoric acid, and 1 mL of 0.6% thiobarbituric acid and incubated at 100°C for 45 min. After the temperature was reduced, 4 mL of n-butanol was added and mixed well. Finally, after centrifugation, the optical absorption of the n-butanol phase was measured at 532 nm [35]. Furthermore, the testis tissue samples were agitated in ice-cold phosphate-buffered saline and then centrifuged at a force of 10,000 g for 15 min. The total antioxidant capacity (TAC) (KTAC-96) and MDA (KMDA96) levels were measured using the colorimetric technique on the liquid part, using kits acquired from Kiazist Company (Iran).

### 2.7. RNA Extraction and Real-Time PCR

After slaughtering the rats, one of the testis tissues from each rat was preserved in a liquid nitrogen (N_2_) tank to maintain the RNA content. Total RNA was extracted using the Kiyanzol isolation reagent from each sample. The concentration and purity of the extracted RNA samples were measured using a NanoDrop (Thermo Fisher Scientific, Waltham, MA, USA), and the integrity of the RNA samples was assessed through horizontal gel electrophoresis. To ensure complete RNA isolation, an additional DNase I (Qiagen) step was included according to the manufacturer's instructions. Subsequently, 1 μg of RNA was used for cDNA synthesis using the QuantiTect Reverse Transcription Kit (Fermentas), following the provided protocol with a final volume of 20 μL. The synthesized cDNAs were stored for use in quantitative real-time polymerase chain reaction (RT-PCR) experiments. RT-PCR analysis was performed using the ABI 7500 equipment (Applied Biosystems Inc., Foster City, CA, USA) to assess the mRNA expressions of *Star*, *Cyp11a1*, and *Hsd17b3* in the testes. The primer sequences for the quantitative RT-PCR analysis are provided in Table 1. Additionally, the melting curve analysis was conducted to evaluate the specificity of the PCR product, and the slope of the standard curve was used to determine the amplification efficiency. Finally, the 2^−ΔΔCt^ method was employed to calculate relative expression, with the housekeeping gene glyceraldehyde 3-phosphate dehydrogenase (GAPDH) serving as the internal reference.

### 2.8. Analysis of Sperm Parameters (Count, Motility, Viability, Morphology)

To determine motility and sperm count, 1 cm of the left cauda epididymis was removed and placed in 5 mL of Hanks solution to establish a good environment for sperm survival in a short length of time.

Sperm were classified into three groups based on the type of movement:

- Progressive movement
- Nonprogressive movement
- Immotile

To count sperms, one drop of the sperm-containing fluid was applied to the neobar lam. The average number of sperms was calculated by counting the number of sperms on each of the four large squares. The total number of extricated sperms from 1 cm of the cauda epididymis was estimated using the formula below:

(1) $$\begin{array}{c} A=B.C.D. \end{array}$$

*A*: The total number of sperms extracted from the cauda epididymis.

*B*: The number of counted sperms per 0.1 mm^3^ of solution.

*C*: Depth factor equals 10.

*D*: Dilution factor = 5000 mm^3^ (sperm from 1 cm of cauda epididymis discharged in 5 mL of solution).

#### 2.8.1. Evaluation of Sperm Viability

Eosin-nigrosin staining was used to determine sperm viability. Eosin and nigrosin were produced using distilled water (both manufactured by Merck in Darmstadt, Germany). One liter of sperm suspension was combined with two volumes of 1% eosin. After 30 s, add an equal volume of nigrosin to the mixture. Create thin smears and inspect them with a light microscope (Nikon E-200, Japan) at a magnification of 40×. In this fashion, viable sperms remained colorless, whereas nonviable sperms turned scarlet [36].

#### 2.8.2. Sperm Morphology Analysis and Estimated Percentage of Abnormal Sperms

The suspension was placed on a slide, stained with 1% eosin Y for 5–10 min, then allowed to dry. Each sample contained between 100 and 200 spermatozoa per rat, and the percentage of aberrant sperm was calculated. Amorphous heads, two heads, merged bodies, two tails, and a normal-shaped head with a damaged or twisted tail were all termed aberrant sperm [37, 38].

### 2.9. Pathological Examinations

The separated testicular tissues were weighed and measured in size before fixation. To prepare the sample pathological blocks, the tissues underwent a dehydration process involving immersion in a series of ascending alcohol concentrations, gradually progressing toward pure alcohol. Xylene was employed to enhance transparency, owing to its ability to dissolve paraffin. Subsequently, the circular pieces of sample slabs were embedded into paraffin, in conformity with the established protocol. Subsequently, sections of 5-μm thickness were obtained via a microtome and subjected to hematoxylin-eosin staining (H&E) for volume assessment of the selected components. Upon the examination of the slides using a microscope (Nikon E200), the modified Johnson method was employed for the calculation of testicular tissue changes (Table 2).

### 2.10. Statistical Analysis

The data were analyzed by the SPSS software (Version 20, IBM Inc., USA). Normality of the data was assessed using the Shapiro–Wilk test. Based on the normality of the data for statistical analysis, one-way analysis of variance (ANOVA) along with Tukey's post hoc test was utilized. A *p* value of less than 0.05 was deemed statistically significant. The results were reported as mean ± standard deviation (SD).

## 3. Results

### 3.1. FBS Level and Body Weight

**Figure 1:**
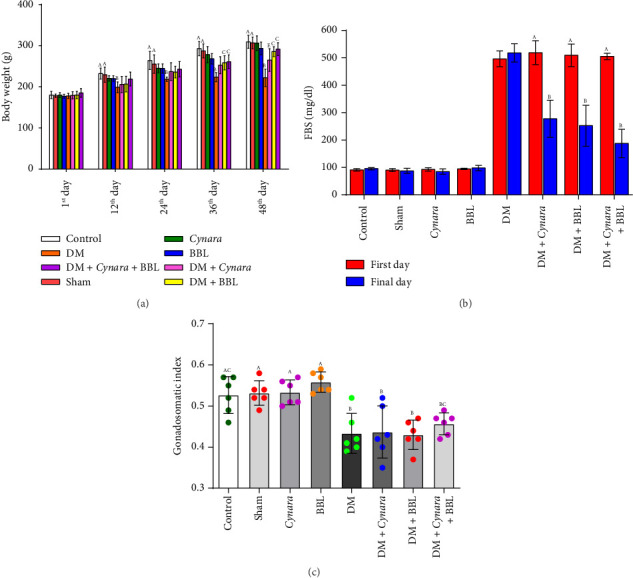
The effects of Cynara and BBL on the following investigated groups' (a) body weight changes throughout 48 days, (b) changes in FBS values between the start and end of the trial, and (c) gonadosomatic index in the following studied groups: Control group (Control), sham group (Sham), healthy group receiving Cynara (Cynara), healthy group receiving BBL (BBL), diabetic group (DM), diabetic group receiving Cynara (DM + Cynara), diabetic group receiving BBL (DM + BBL), diabetic group receiving Cynara + BBL (DM + Cynara + BBL). Data are shown as mean ± SD. In the figures, the presence of at least one identical uppercase letter in the columns indicates no significant difference between groups. Conversely, the absence of similar letters in the columns signifies a significant difference at the level of *p* < 0.05.

According to Figure 1(a) the mean body weight of the diabetic group demonstrated a significant decrease (*p* < 0.001) in comparison to the healthy groups after the 48-day treatment period. Conversely, the treatment with *Cynara* (*p* = 0.004) and BBL (*p* < 0.001), both separately and as the combined treatment of BBL and *Cynara* (*p* < 0.001), resulted in a significant increase in the body weight of diabetic rats when compared to the diabetic group. The changes in body weight were monitored over the duration of a 48-day treatment, as shown in Figure 1(b) (1st, 12th, 24th, 36th, and 48th days). It is important to note that the rats' weights are the same on the first day with no noticeable variations. When compared to healthy rats, diabetic animals display significantly diminished during these times. When compared to the diabetic group, the mean body weight of the treated groups (DM + *BBL*, DM + *BBL* + *Cynara*) increased significantly on the 36th and 48th days. On the 48th day, there were notable variations seen between DM + *Cynara* and DM + *BBL*, DM + *BBL* + *Cynara*.

The diabetic group of rats exhibited a significant increase in mean FBS concentration when compared to the control and sham groups (*p* < 0.001) after the 48-day period. However, the administration of *Cynara* and BBL separately, as well as the combined treatment of BBL and *Cynara* at the specified concentrations, led to a significant reduction in FBS concentration in diabetic rats (*p* < 0.001) Figure 1(b). Furthermore, the FBS level in the Control, Sham, *Cynara*, *BBL*, and DM rats did not significantly change between the 1st and 48th days of treatment, but in the diabetic-treated groups (DM + *Cynara* and DM + *BBL*, DM + *BBL* + *Cynara*), the FBS level significantly decreased from the first day (Figure 1(b)).

Gonadosomatic index is a value that expresses the proportion of testis weight to body weight. As seen in Figure 1(c), the gonadosomatic index of the diabetic rats was much lower than that of the control group. The results revealed no significant difference (*p* > 0.05) between the BBL and *Cynara* treatments, when given separately and in combination, and the DM group.

### 3.2. Hydroalcoholic Extract of *Cynara scolymus L* Combined With Probiotic BBL Ameliorate Antioxidant Capacity in Diabetic Rats

**Figure 2:**
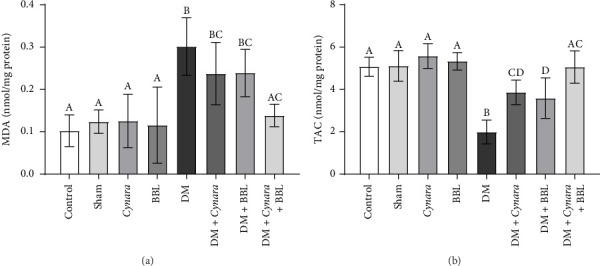
MDA (a) and TAC (b) level in testis tissue of treated groups. Control group (Control), sham group (Sham), healthy group receiving Cynara (Cynara), healthy group receiving BBL (BBL), diabetic group (DM), diabetic group receiving Cynara (DM + Cynara), diabetic group receiving BBL (DM + BBL), diabetic group receiving Cynara + BBL (DM + Cynara + BBL). Data are shown as mean ± SD. The presence of at least one identical uppercase letter in the columns indicates no significant difference between groups. Conversely, the absence of similar letters in the columns signifies a significant difference at the level of *p* < 0.05.

Figure 2 shows a notable decrease in TAC levels in testis of diabetic rats, but significant improvement was observed in the treated groups, particularly in the DM + Cynara + BBL group. Contrarily, the level of MDA, which serves as an indicator of lipid peroxidation, significantly increased in the treated groups with diabetes. However, it was decreased in the DM + Cynara + BBL group, and it was not different from the control group. According to the data presented in Table 3, the DM group exhibited a notably higher serum level of MDA, serving as an indicator of lipid peroxidation in comparison to the control and sham groups. Treatment with *Cynara* and BBL separately, as well as the combined treatment of BBL and *Cynara*, led to a significant reduction in the MDA serum level in diabetic rats when compared to the DM group. However, the MDA serum level in the treated diabetic groups did not reach the level observed in the nondiabetic groups.

### 3.3. Hydroalcoholic Extract of *Cynara scolymus L* Combined With Probiotic BBL Improved Serum Sexual Hormones in Diabetic Rats

As depicted in Table 3, the LH, FSH, and testosterone levels demonstrated a notable decrease in the DM group as compared to the control, sham, *Cynara*, and BBL groups (*p* < 0.001). Treatment with *Cynara* and BBL and their combination in diabetic rats led to a significant increase in the testosterone levels in comparison to the DM group at the level of (*p* = 0.030), (*p* = 0.013), and (*p* < 0.001), respectively. Furthermore, the treatments with *Cynara* and BBL separately and their combination in diabetic rats resulted in a greater elevation of the LH hormone (*p* < 0.001). Moreover, the treatment with BBL and *Cynara* + BBL in diabetic rats resulted in a significant increase in FSH hormone but not Cynara (*p* < 0.001). However, the observed increment in LH and FSH levels within the combination treatment group was not deemed statistically significant when compared to the *Cynara* and BBL groups in diabetic rats. Conversely, the testosterone level exhibited a significant increase in the combination treatment group in comparison to the DM + *Cynara* and BBL groups in diabetic rats (*p* < 0.001).

### 3.4. Effects of Hydroalcoholic Extract of *Cynara scolymus L Combined* With Probiotic BBL *on Star*, *Cyp11a1*, and *Hsd17b3* mRNA Expression Levels

**Figure 3:**
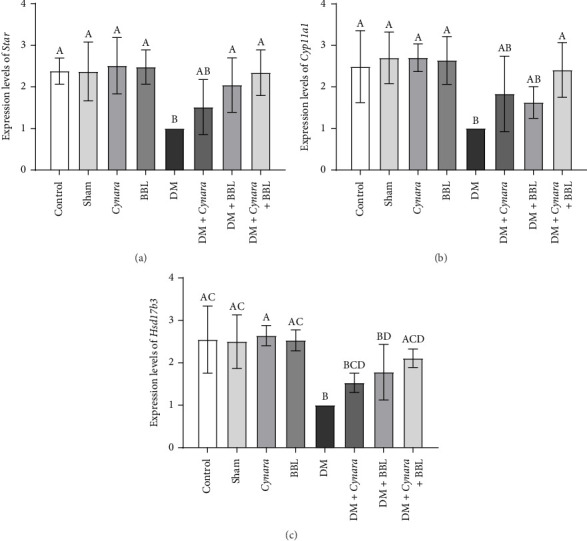
The mRNA expression levels of (a) *Star*, (b) *Cyp11a1*, and (c) *Hsd17b3* of the following studied groups: Control group (Control), sham group (Sham), healthy group receiving Cynara (Cynara), healthy group receiving BBL (BBL), diabetic group (DM), diabetic group receiving Cynara plant (DM + Cynara), group of diabetics receiving BBL (DM + BBL), diabetic group receiving Cynara + BBL (DM + Cynara + BBL). Data are shown as mean ± SD. The presence of at least one identical uppercase letter in the columns indicates no significant difference between groups. Conversely, the absence of similar letters in the columns signifies a significant difference at the level of *p* < 0.05.

As depicted in Figure 3, the mRNA expressions of the *Star* gene exhibited a significant decrease in the DM group compared to the control and sham groups (*p* = 0.002), just as *Cyp11a1* gene expression decreased significantly in the DM group compared to the control and sham group (*p* = 0.003 and *p* = 0.001, respectively). In the diabetic group, a definite decrease in *Hsd17b3* gene expression was observed compared to the control and sham groups (*p* < 0.001). Treatment of diabetic rats with *Cynara* alone resulted in a nonsignificant increase in the mRNA genes expression levels compared to the DM group. Conversely, BBL treatment led to a significant increase in only the *Star* mRNA expression level (*p* = 0.038). No significant effect was observed for the other two examined genes (*Cyp11a1*, *Hsd17b3*). Notably, treatment of the diabetic group with the combination of *Cynara* and BBL yielded a significant increase in the mRNA levels of *Star*, *Cyp11a1*, and *Hsd17b3* genes in the testes of diabetic rats (*p* = 0.003, *p* = 0.006, and *p* = 0.003, respectively).

### 3.5. Effects of *Cynara scolymus* Hydroalcoholic Extract and Probiotic BBL on Sperm Parameters

There were no variations in any of the sperm parameters across the healthy groups (control, sham, Cynara, and BBL), as Table 4 indicates. The percentage of immotile sperm and aberrant sperm morphology increased significantly (*p* < 0.001) in the diabetes groups when compared to the other healthy groups, according to the data. Nevertheless, these characteristics decreased after diabetic rats were treated. Sperm count and percentage of progressive sperm movement were significantly lower in the diabetic groups compared to the healthy groups (*p* < 0.001). The sperm count and the percentage of progressive sperm movement were significantly highter in the diabetic groups treated with Cynara, BBL, and Cynara + BBL (*p* < 0.01) than in the diabetic rats. The findings demonstrated that, in comparison to the other healthy groups, diabetic rats had a higher nonprogressive sperm movement (*p* < 0.001). Nevertheless, no variations were observed between the diabetic one and the DM + Cynara, DM + BBL, and DM + Cynara + BBL groups. On the other hand, the percentage of nonviable sperm in diabetic rats was much lower than in healthy rats (*p* < 0.001), but it increased dramatically in diabetic rats treated with a combination of Cynara and BBL.

### 3.6. Effects of *Cynara scolymus* Hydroalcoholic Extract and Probiotic BBL on Testicular Weight, Volume, Leydig Cell Count, Modified Johnson's Score, and Histological Alterations

Table 5 shows the changes in testes' weight and size in each experimental group. The testis' weight and volume in the diabetic group showed a significant decrease compared to the control group (*p* < 0.001). The average weight of the testis in all the diabetic groups receiving Cynara, BBL, and Cynara + BBL showed a significant increase compared to the diabetic group (*p* = 0.025, *p* < 0.001, and *p* < 0.001, respectively). No significant difference between the testis' weight and volume in the control group, sham, and healthy groups receiving Cynara and BBL was observed. The average weight of the testes in all diabetic groups under treatment showed a significant decrease compared to the control, sham, and healthy groups receiving Cynara and BBL at the level (*p* < 0.001). The average testicular volume in all diabetic groups receiving the Cynara, BBL, and Cynara + BBL showed a significant increase compared to the diabetic group (*p* = 0.038, *p* < 0.001, and *p* < 0.001, respectively). The average testicular volume in all diabetic groups under treatment showed a significant decrease compared to the control, sham, and healthy groups receiving Cynara and BBL at the level (*p* < 0.001). The average testicular volume in the diabetics treated with Cynara + BBL showed a significant increase in level compared to the diabetic group receiving Cynara (*p* = 0.028). According to Table 5, the number of Leydig cells substantially dropped in diabetic rats, but it significantly increased in the treated groups, particularly in the DM + Cynara + BBL group. The difference between this group and the control group was not significant.

**Figure 4:**
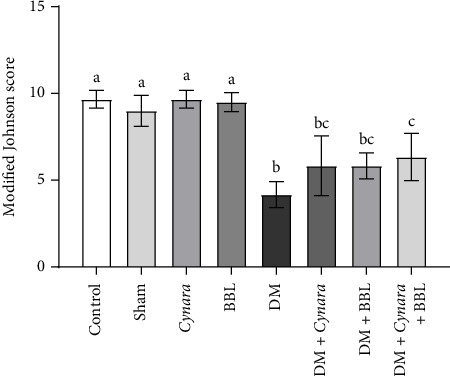
The average of modified Johnson's score in the following studied groups: Control group (Control), sham group (Sham), healthy group receiving Cynara (Cynara), healthy group receiving BBL (BBL), diabetic group (DM), diabetic group receiving Cynara plant (DM + Cynara), group of diabetics receiving BBL (DM + BBL), diabetic group receiving Cynara + BBL (DM + Cynara + BBL). The Johnson score in the diabetic group showed a significant decrease compared to the control and sham groups. The results showed a significant increase in the average of the Johnson score in the diabetic group receiving Cynara + BBL combination compared to the diabetic group at the level. The presence of at least one identical lowercase letter in the columns indicates no significant difference between groups. Conversely, the absence of similar letters in the columns signifies a significant difference at the level of *p* < 0.05. Data are shown as mean ± SD.

According to the data in Figure 4, the mean of the modified Johnson's scoring in the diabetic group demonstrated a significant decrease compared to the control and sham groups (*p* < 0.001). The results indicated a noteworthy increase in the mean of the modified Johnson's scoring in the diabetic group treated with the combination of *Cynara* and BBLin comparison to the diabetic group treated with either of the two separately (*p* = 0.009). However, no significant difference was observed between the control, sham, and nondiabetic groups that received either *Cynara* or BBL. Additionally, there was no significant difference between the diabetic group treated with *Cynara* and the diabetic group treated with BBL in comparison to the diabetic group (*p* < 0.001). The mean of the modified Johnson's scoring in the diabetic group treated with *Cynara* + BBL demonstrated a significant decrease compared to the control group, sham group, and nondiabetic groups that received either *Cynara* or BBL separately (*p* ≤ 0.001). No significant difference was found among the remaining groups (*p* < 0.05).

**Figure 5:**
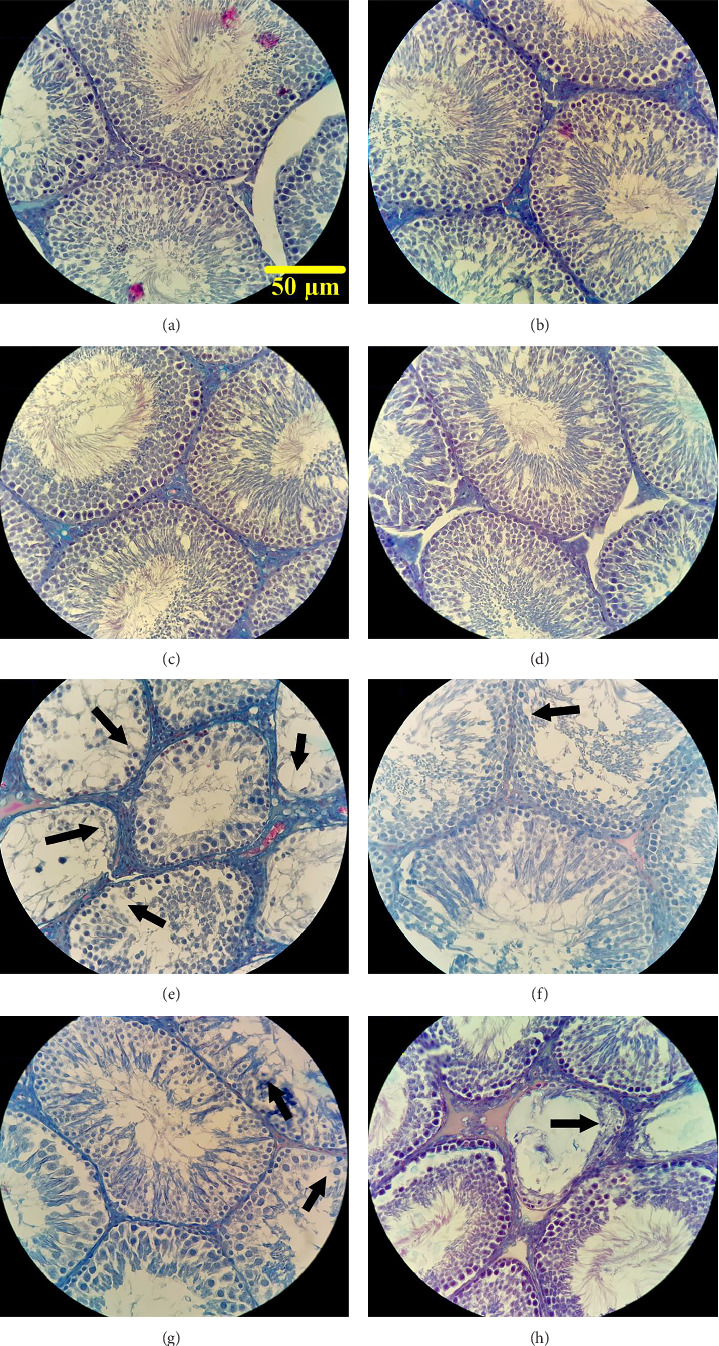
Photomicrograph of the histopathological changes in testicular tissue with H&E staining, magnification 400x of the following groups: (a) control group, (b) sham group, (c) healthy group receiving *Cynara*, (d) healthy group receiving BBL, (e) diabetic group atrophy and shrinkage of the tubules, due to the disintegration of the germinal epithelium, a severe decrease in the number of germ cells and the presence of empty spots inside the tubules (arrow sign) indicate the destructive effects of diabetes on the testicular tissue, (f) diabetic group receiving *Cynara*, (g) diabetics group receiving BBL, and (h) diabetic group receiving *Cynara* + BBL. In the f, g, and h groups, a significant increase in the sexual lineage cell numbers was observed, although it was not as high as the control group. Also, the presence of cell-free tubules and shrinkage of the seminiferous tubules (arrow sign) were still observed in some parts of the testicular tissues (x40).

According to Figure 5, the control group, sham group, and healthy groups receiving *Cynara* and BBL exhibited normal tubules and germ cells without any pathological damage. As measured by Johnson's scoring system, these groups received scores higher than 9, indicating completely regular lumens and normal cell types, including spermatogonia, spermatocytes, round and elongated spermatids (SPTs), Sertoli, and Leydig cells. Active spermatogenesis was observed with a normal thickness of the germinal epithelium and no congestion or edema in the connective tissue. In contrast, the diabetic group displayed tubular atrophy and shrinkage, disintegration of the germinal epithelium, severe reduction in the number of germ cells, and the presence of empty spaces within the tubules (indicated by arrow signs), signifying the detrimental effects of diabetes on testicular tissue. Furthermore, this group exhibited a significant decrease in germinal epithelium thickness and congestion of blood vessels in the interstitial tissue. In the diabetic groups treated with *Cynara* extract and BBL in combination, a notable increase in the number of germ cells was observed, although it did not reach the level seen in the control group. Some areas of the testicular tissue still showed the presence of cell-free tubules and tubular shrinkage (indicated by arrow signs).

## 4. Discussion

The results of the current study demonstrate the promising therapeutic potential of the hydroalcoholic extract of *Cynara scolymus* (*Cynara*) and BBL probiotic treatment, separately and in combination, in mitigating the detrimental effects of diabetes on male reproductive function. The findings underscore the notable benefits of these treatments in ameliorating various physiological parameters, including FBS levels, serum MDA levels, and serum sexual hormone level, particularly LH, FSH, and testosterone. Our investigation revealed that the combination therapy of *Cynara* and BBL led to greater improvements in certain parameters compared to individual treatments. Specifically, this combination treatment demonstrated superior effects in enhancing the serum levels of LH, FSH, and testosterone, as well as in elevating the mRNA expressions of key testicular steroidogenesis enzymes, including *Star*, *Cyp11a1*, and *Hsd17b3*. Histopathological assessments confirmed the protective role of *Cynara* and BBL in preserving testicular tissue integrity, preventing tubular atrophy and shrinkage, and promoting the restoration of normal germ cell populations.

Streptozotocin (STZ)-induced diabetes promotes excessive ROS production in pancreatic β cells, causing DNA damage and cell destruction [39]. Diabetic rats exhibit elevated MDA levels, reflecting increased ROS in serum and tissues [40]. This oxidative stress, along with elevated FBS, correlates with reduced pituitary hormones LH and FSH, leading to impaired testicular function, including decreased *Star* expression and lower testosterone levels [5, 41]. Herbal medicines rich in antioxidants have been proposed to mitigate free radical damage in diabetes [42]. Moreover, the intestinal microbiota plays a key role in physiological processes such as gut permeability, systemic inflammation, hormone secretion, and energy metabolism, influencing the progression of Type 2 diabetes [43, 44]. Targeting the gut microbiome has thus emerged as a potential strategy for diabetes management [45]. In our study, we implemented a symbiotic approach combining *Cynara* extract and BBL probiotics to investigate potential synergistic effects on diabetic outcomes.

Our findings showed that all treated diabetic groups exhibited lower FBS compared with untreated diabetic rats, with the *Cynara* + BBL group showing the greatest reduction. These results align with previous studies demonstrating the antidiabetic effects of probiotics and herbal extracts, though mechanisms remain incompletely understood. *Cynara* has been reported to improve glycemic control by enhancing insulin sensitivity and inhibiting α-amylase activity, thereby reducing glucose absorption [46, 47]. Inulin from *Cynara*, with its high degree of polymerization, also modulates gut microbiota and exerts bifidogenic, antioxidant, and fiber-related effects that support diabetes control [48–50]. Similarly, probiotics have shown beneficial roles in lowering blood glucose and preventing complications by improving gut microbiota composition and generating secondary metabolites [51–53]. According to our data, a noticeable decline in body weight was observed in rats with induced diabetes, whereas treatment with *Cynara*, BBL, and their combination effectively prevented the loss of body weight in these diabetic rats. Weight loss in diabetic rats is attributed to factors such as tissue protein breakdown, lipid catabolism, and polyuria, which arise due to an impaired ability to utilize carbohydrate sources. This metabolic limitation shifts energy reliance to fat reserves, leading to structural protein degradation and a subsequent reduction in body weight. In treated groups, the prevention of weight loss was partially attributed to the improvement in blood glucose levels. Similarly, a previous study found that diabetic rats treated with *Cynara* extract were able to regain the weight lost due to diabetes. A previous study has revealed the effect of 5 weeks oral administration of probiotics containing “*L. rhamnosus L12*, *L. acidophilus*, *L. plantarum HM218749*, *B. animalis* subsp. *lactis LPL-RH*, *B. longum* subsp. *longum BAMA-B05/BAu-B1024*” resulted in a slightly gaining weight in STZ-induced diabetes rats [54]. Nonetheless, another investigation found no significant body weight increase in STZ-induced diabetic rats after 8-week treatment with multistrain probiotics [55]. Reduced testosterone secretion has also been identified as a factor contributing to muscle mass loss in diabetic rats, along with the metabolic shift toward fat utilization and subsequent protein degradation [56]. Similarly, *Cynara* extract has been shown to restore body weight in diabetic rats [46].

In our study, diabetic rats showed a significant increase in MDA levels and a decrease in TAC compared with control and sham groups. Treatment with *Cynara*, BBL, and their combination effectively reduced MDA and increased TAC, highlighting their strong antioxidant potential. These results align with prior findings demonstrating that BBL can decrease hepatic MDA via NRF2 pathway regulation [29, 57], and that BBL combined with *L. acidophilus* can suppress lipid peroxidation and remove MDA byproducts [58]. Similarly, *Cynara* extract, rich in cynarin, luteolin, and chlorogenic acid, can protect pancreatic β-cells from oxidative damage, supporting the observed improvement in oxidative stress markers [59].

Diabetes is known to disrupt Leydig cell function through increased proinflammatory cytokines and hormonal imbalances, leading to suppression of the hypothalamus-pituitary axis and reduced plasma gonadotropins and testosterone [60]. In the current study, diabetic rats exhibited a significant decrease in LH, FSH, and testosterone compared to control and sham groups. Treatment with hydroalcoholic *Cynara* extract, Bifidobacterium (BBL), and their combination significantly restored these hormone levels, particularly LH and FSH in the BBL and *Cynara* + BBL groups. These effects likely result from antioxidant and antilipid peroxidation actions that protect gonadotropic cells and support Leydig cell steroidogenesis. The observed reduction in testosterone in diabetes is consistent with increased oxidative stress and imbalances between prooxidants and antioxidants [1] Enhancing the gut microbiome alongside antioxidant supplementation may further improve testosterone secretion and represent a potential strategy for mitigating diabetes-associated reproductive dysfunction [61].

Both Type 1 and Type 2 diabetes negatively impact male fertility, affecting sperm motility, DNA integrity, and seminal plasma components [4]. In our study, single and combined administration of *Cynara scolymus* extract and BBL in diabetic rats demonstrated potent antioxidant and lipid peroxidation inhibitory effects, which likely contributed to improvements in reproductive markers via antidiabetic, antioxidative, and hypolipidemic mechanisms. Previous research has highlighted the complexity of spermatogenesis, in which diploid spermatogonia differentiate into haploid spermatozoa through physiological, biochemical, and morphological modifications [62, 63]. We also examined key steroidogenic genes. As reported in prior studies, STZ-induced diabetes decreased *Star* expression, consistent with our findings, whereas *Cyp11a1* and *Hsd17b3* mRNA levels showed no significant changes. These results suggest that *Cynara* and BBL may primarily exert protective effects on early steroid transport rather than on downstream androgen synthesis [64]. In this study, diabetic rats showed a significant reduction in the expression of *Star*, *Cyp11a1*, and *Hsd17b3* compared to control and sham groups. Oxidative stress has been reported to impair key steroidogenic molecules in Leydig cells, including *Star*, *Hsd17b3*, and *Cyp11a1*, leading to reduced testosterone synthesis [65, 66]. In the current study, the level of *Star* gene expression in diabetic rats treated with BBL and the combination therapy was significantly increased compared to untreated diabetic rats. Moreover, the expression of *Hsd17b3* and *Cyp11a1* was notably elevated only in the combination treatment group. Recent research indicates that Type 1 diabetes (T1D) reduces steroidogenic enzyme protein levels in rat testicular somatic and germ cells via oxidative stress mediated by the NRF2/NLRP3 pathway. This inflammation and oxidative stress impair steroid hormone synthesis and contribute to testicular dysfunction in men with T1D. Lower levels of N-cadherin (N-CAD), zonula occludens-1 (ZO-1), open colony network (OCN), and connexin 43 (CX43) proteins have been reported in T1D rat testes. Diabetes also affects testicular VANGL planar cell polarity protein 2 (VANGL2), a protein in the apical ectoplasmic specialization (ES), as well as steroid receptor coactivator (Src) and focal adhesion kinase (FAK) activation. VANGL2 regulates the spatial and temporal organization of actin microfilaments and microtubules, maintaining blood–testis barrier (BTB) integrity and facilitating SPT passage toward the lumen [67]. Consistent with our findings, steroidogenesis-related genes are downregulated in male rats with streptozotocin (STZ)-induced diabetes, contributing to reduced fertility. Metformin treatment has been reported to mitigate these effects by enhancing steroidogenic gene expression and improving reproductive function in diabetic rats [68]. Similarly, the natural compound umbelliferone protects testicular tissue in Type 2 diabetic rats from oxidative stress, promotes steroidogenesis, and increases expression of peroxisome proliferator-activated receptor gamma (PPARγ), a key regulator of inflammation and lipid metabolism, thereby supporting testicular and overall reproductive health [69]. Histopathological analysis revealed a significant reduction in testicular weight, size, and modified Johnson score in diabetic rats compared to control and sham groups. Treatment with *Cynara*, BBL, and their combination significantly improved testicular weight and volume, with the combination therapy also enhancing the modified Johnson score, although not to levels observed in healthy rats. Diabetic rats exhibited a marked decrease in Sertoli cells and disrupted germ cell populations, explaining the reduction in spermatogonial cells, spermatocytes, and SPTs, which contributes to decreased testicular weight and volume. These findings are consistent with previous reports showing that STZ-induced diabetes reduces serum testosterone, testicular weight, tubule diameter, and total sperm count in rats [70]. Bifidobacterium probiotics have been reported to increase sperm count in diabetic rats by lowering blood glucose and suppressing proinflammatory mediators such as lipopolysaccharides [52]. Similarly, garlic extract has shown preventive and therapeutic effects on diabetes-induced testicular damage, improving seminiferous tubule diameter through its antioxidant properties [71], and garlic-derived inulin as a prebiotic ameliorates reproductive complications in Type 2 diabetic mice [72]. In line with these findings, our study demonstrates that both *Cynara* and BBL treatments significantly alleviate diabetic symptoms and improve reproductive parameters, including testicular atrophy in STZ-induced diabetic rats, with the combination therapy showing superior effects.

Despite these promising results, several limitations should be noted. Assessment of cellular health would benefit from apoptosis-associated staining such as TUNEL assay. Although mRNA expression of *Star*, *Cyp11a1*, and *Hsd17b3* was measured via qRT-PCR, further validation using Western blot and quantification of protein levels in relevant signaling pathways is recommended. Additional studies are also needed to clarify underlying mechanisms and evaluate the long-term safety and efficacy of these treatments in clinical settings.

## 5. Conclusion

In sum, our findings suggest that *Cynara* and BBL may exert beneficial effects in mitigating the adverse impact of diabetes on male reproductive function and fertility. While these natural and probiotic-based treatments show promise as potential complementary or alternative therapeutic options, further studies are required to validate these effects and to clarify the underlying mechanisms before clinical application can be considered.

## Figures and Tables

**Table 1:** The sequence of designed primers.

Primer	Sequences (5′ -> 3′)	PCR product length (bp)
STAR:F	AGATGAAGTGCTAAGTAAG	150
STAR:R	TTGATTTCCTTGACATTTG	

GAPDH:F	AAAGAGATGCTGAACGGGCA	100
GAPDH:R	ACAAGGGAAACTTGTCCACGA	

Cyp11a1:F	AAAGTATCCGTGATGTGGG	112
Cyp11a1:R	TTTCTGGGCATAGTTGAGC	

Hsd17b3:F	ATTACCTCCGTAGTCAAGA	176
Hsd17b3:R	TATTCCACATTCAAAGCCT	

**Table 2:** Evaluation of the maturity of the spermatogenesis process based on Johnson's scoring.

Score description	
10	Complete spermatogenesis and perfect tubule
9	Many late spermatids present but disorganized tubular epithelium
8	Only a few late spermatids
7	No late spermatids but many early spermatids
6	Few early spermatids, arrest of spermatogenesis at the spermatid stage, disturbance of spermatid differentiation
5	No spermatids, many spermatocytes
4	Few spermatocytes, arrest of spermatogenesis at the primary spermatocyte stage
3	Only spermatogonia
2	No germ cells, Sertoli cells only
1	No germ cells or Sertoli cells, tubular sclerosis

**Table 3:** The results related to the average concentration of serum LH, FSH, testosterone, and malondialdehyde (MDA).

Group	LH (mIU/mL)	FSH (mIU/mL)	Testosterone (ng/mL)	MDA (nmol/mL)
Control	23.45 ± 3.03^a^	26.45 ± 2.64^a^	0.953 ± 0.04^a^	0.031 ± 0.008^a^
Sham	23.45 ± 2.62^a^	26.12 ± 2.53^a^	0.954 ± 0.08^a^	0.024 ± 0.009^a^
*Cynara*	23.12 ± 3.68^a^	25.87 ± 2.08^a^	0.973 ± 0.03^a^	0.29 ± 0.018^a^
BBL	23.52 ± 2.47^a^	26.45 ± 2.72^a^	0.957 ± 0.06^a^	0.031 ± 0.017^a^
DM	10.33 ± 2.58^b^	12.00 ± 6.42^b^	0.382 ± 0.04^b^	1.19 ± 0.33^b^
DM + *Cynara*	14.67 ± 1.86^bc^	15.00 ± 5.93^bc^	0.521 ± 0.07^d^	0.62 ± 0.22^c^
DM + BBL	18.83 ± 1.17^ac^	19.66 ± 3.43^ac^	0.533 ± 0.09^d^	0.48 ± 0.19^c^
DM + *Cynara* + BBL	19.00 ± 2.84^ac^	20.83 ± 2.64^ac^	0.717 ± 0.11^c^	0.42 ± 0.25^c^
*p* value	< 0.0001	< 0.0001	< 0.0001	< 0.0001

*Note:* Data are shown as mean ± SD. Columns sharing at least one identical lowercase letter (a, b, c, d) indicate no significant differences between the corresponding groups. Columns without shared lowercase letters signify a significant difference at the level of *p* < 0.05.

**Table 4:** The effect of *Cynara scolymus hydroalcoholic* extract and probiotic *Bifidobacterium longum* on sperm parameters.

Group	Sperm count (× 10^6^)	Sperm progressive movement (%)	Nonprogressive movement (%)	Immotile sperm (%)	Abnormal sperm morphology (%)	Nonviability sperm (%)
Control	12.97 ± 0.95^a^	80.51 ± 5.40^a^	14.41 ± 4.12^a^	5.07 ± 2.72^a^	7.83 ± 1.60^ac^	6.50 ± 1.64^ac^
Sham	13.23 ± 0.93^a^	78.86 ± 2.79^a^	14.17 ± 2.89^a^	6.96 ± 2.25^a^	6.83 ± 0.98^ac^	6.67 ± 1.63^ac^
Cynara	14.83 ± 0.73^a^	81.69 ± 1.48^a^	13.78 ± 1.52^a^	4.52 ± 1.92^a^	6.17 ± 1.60^a^	4.00 ± 1.41^a^
BBL	14.12 ± 2.01^a^	82.17 ± 6.85^a^	12.67 ± 6.89^a^	5.17 ± 2.04^a^	8.00 ± 1.67^ac^	4.00 ± 2.28^ac^
DM	4.05 ± 0.74^b^	6.28 ± 1.81^b^	30.95 ± 4.08^b^	62.76 ± 3.80^b^	54.67 ± 16.03^b^	74.17 ± 8.28^b^
DM + Cynara	6.90 ± 0.93^c^	20.23 ± 8.34^d^	35.34 ± 5.13^b^	44.43 ± 13.05^d^	23.83 ± 4.17^d^	53.83 ± 24.75^cb^
DM + BBL	8.80 ± 1.33^c^	37.17 ± 8.61^c^	35.17 ± 8.51^b^	27.67 ± 7.53^c^	24.67 ± 7.89^d^	56.00 ± 12.90^cb^
DM + Cynara + BBL	8.84 ± 1.88^c^	45.54 ± 7.78^c^	31.12 ± 2.27^b^	23.34 ± 6.16^c^	16.67 ± 7.94^cd^	52.67 ± 11.36^c^

*Note:* The results have been presented as mean ± SD. The presence of at least one identical lowercase letter in the columns indicates no significant difference between groups. Conversely, the absence of similar letters in the columns signifies a significant difference at the level of *p* < 0.05.

**Table 5:** The results related to the testis weight (g) and testis volume (mm^3^) and the number of Leydig cells.

Group	Average testis weight (g)	Average testis volume (mm^3^)	Leydig cells (× 10^6^)
Control	1.63 ± 0.085^a^	1548.67 ± 92.58^a^	40.50 ± 4.92^a^
Sham	1.63 ± 0.027^a^	1532.5 ± 31.98^a^	39.16 ± 2.56^a^
*Cynara*	1.63 ± 0.096^a^	1548.67 ± 86.08^a^	39.99 ± 4.84^a^
BBL	1.64 ± 0.05^a^	1567.17 ± 70.43^a^	39.88 ± 3.90^a^
DM	0.96 ± 0.14^b^	871 ± 132.23^b^	10.81 ± 2.87^b^
DM + *Cynara*	1.14 ± 0.093^d^	1042.17 ± 63.56^d^	26.71 ± 13.63^c^
DM + BBL	1.23 ± 0.095^cd^	1127.67 ± 84.06^cd^	27.47 ± 1.91^c^
DM + *Cynara* + BBL	1.32 ± 0.11^c^	1219.66 ± 116.86^c^	34.73 ± 4.65^ac^

*Note:* Data are shown as mean ± SD. The presence of at least one identical lowercase letter in the columns indicates no significant difference between groups. Conversely, the absence of similar letters in the columns signifies a significant difference at the level of *p* < 0.05.

## Data Availability

This article contains all data created and examined throughout this investigation. The corresponding author will provide datasets used or analyzed during the current work upon reasonable request.

## Nomenclature

BBL: *Bifidobacterium longum*
*Cyp11a1*: Cytochrome P450 family 11 subfamilies a polypeptide 1
ELISA: Enzyme-linked immunosorbent assay
FBS: Fasting blood sugar
FSH: Follicle-stimulating hormone
*Hsd17b3*: 17β-Hydroxysteroid dehydrogenase-3
LH: Luteinizing hormone
MDA: Malondialdehyde
ROS: Reactive oxygen species
*Star*: Steroidogenic acute regulatory protein
STZ: Streptozotocin
